# Unraveling the Role of Inflammation in the Pathogenesis of Diabetic Kidney Disease

**DOI:** 10.3390/ijms20143393

**Published:** 2019-07-10

**Authors:** Keiichiro Matoba, Yusuke Takeda, Yosuke Nagai, Daiji Kawanami, Kazunori Utsunomiya, Rimei Nishimura

**Affiliations:** 1 Division of Diabetes, Metabolism, and Endocrinology, Department of Internal Medicine, The Jikei University School of Medicine, Tokyo 105-8461, Japan; 2 Department of Endocrinology and Diabetes Mellitus, Fukuoka University School of Medicine, Fukuoka 814-0180, Japan; 3 Center for Preventive Medicine, The Jikei University School of Medicine, Tokyo 105-8461, Japan

**Keywords:** diabetic kidney disease, diabetic nephropathy, inflammation, signaling cascade

## Abstract

Diabetic kidney disease (DKD) remains the leading cause of end-stage renal disease (ESRD) and is therefore a major burden on the healthcare system. Patients with DKD are highly susceptible to developing cardiovascular disease, which contributes to increased morbidity and mortality rates. While progress has been made to inhibit the acceleration of DKD, current standards of care reduce but do not eliminate the risk of DKD. There is growing appreciation for the role of inflammation in modulating the process of DKD. The focus of this review is on providing an overview of the current status of knowledge regarding the pathologic roles of inflammation in the development of DKD. Finally, we summarize recent therapeutic advances to prevent DKD, with a focus on the anti-inflammatory effects of newly developed agents.

## 1. Introduction

The pandemic of diabetes has become a global health burden. Despite accumulating evidence supporting the prevention of obesity and related metabolic disorders, this knowledge has not translated into action that strongly reduces the prevalence of diabetes, especially in middle- and low-income countries. The International Diabetes Federation (IDF) estimates that diabetes affects 425 million people globally, and the number of diabetic patients will increase to 630 million in 2045.

Such an increase also means that the prevalence of diabetic kidney disease (DKD) continues to rise. DKD is the leading cause of end-stage renal disease (ESRD) and is associated with increased mortality due to cardiovascular disease in subjects with diabetes. Evidence is mounting that albuminuria is not only a hallmark of DKD but also an independent risk factor of coronary disease. The UK Prospective Diabetes Study (UKPDS) demonstrated that annual cardiovascular mortality rates increase to 3%, 4.6%, and 19.2% with the progression to microalbuminuria, macroalbuminuria, and renal failure, respectively [1]. Indeed, patients with DKD are more likely to die from coronary disease than to ever reach ESRD. Due to its high morbidity and mortality, the socioeconomic costs of DKD are staggering. It is therefore urgent that we establish effective and safe therapeutic strategies against DKD in order to improve the prognosis of diabetic patients. To this end, a detailed understanding of the molecular mechanisms that drive DKD is required.

The current understanding of the mechanisms responsible for the progression of DKD recognizes the involvement of metabolic abnormalities (e.g., hyperglycemia, hypertension, and dyslipidemia), hemodynamic changes, renin–angiotensin system (RAS) activation, and oxidative stress. While several approaches have been clinically implemented in order to slow the acceleration of DKD, the current management of DKD is insufficient, both for preventing DKD and for halting its progression.

Given the limitations of therapeutic regimens for inhibiting DKD, there has been an ongoing effort to elucidate the molecular basis responsible for the renal damage and to develop novel drugs. Recent research is characterized by a variety of investigations exploring the inflammatory regulators of DKD. Circulating levels of inflammatory mediators and macrophage infiltration into renal tissue have been found to be increased in both animal models and patients with DKD. Furthermore, adhesion molecules and chemokines are upregulated in diabetic kidneys. These findings highlight the importance of inflammatory mechanisms in facilitating renal damage in the setting of diabetes.

We herein review the inflammatory mediators of diabetes and their contribution to renal injury and describe the potential of anti-diabetic medications as well as drug candidates for inhibiting renal inflammation in the progression of DKD.

## 2. Inflammatory Mediators in Diabetic Kidney Disease (DKD)

Low-grade inflammation is defined as an activation of the innate immune system response. This is clinically considered an increase in circulating levels of pro-inflammatory cytokines and other mediators that activate the immune system. Chronic low-grade inflammation plays a causal role in the progression of obesity and insulin resistance. Deficits in specific genes critical for inflammation are known to be protective against the initiation and development of metabolic diseases in animal models. Therefore, inflammation is considered to be a trigger rather than a result of chronic underlying conditions.

Inflammation is increasingly postulated to be central to the progression of atherogenic changes and microvascular complications in the setting of diabetes [2]. For example, the activation of inflammatory pathways, especially E-selectin and other adhesion molecules, is linked to the pathogenesis of diabetic retinopathy [3]. These adhesion mediators stimulate the migration of retinal capillary endothelium and angiogenesis in the progression of diabetic retinopathy. In addition, evidence of the role of inflammatory signals in the development of diabetic neuropathy has been shown in experimental models of diabetes [4,5] and in clinical studies [6,7]. Diabetic patients with painful peripheral neuropathy have higher levels of inflammatory markers than subjects without pain, and the elevation of interleukin (IL)-1, IL-6, and tumor necrosis factor α (TNF-α) are correlated with the progression of degenerative changes in the peripheral nerves. The expression of nuclear factor-2 erythroid related factor 2 (Nrf2), a regulator of endogenous anti-oxidant production, is decreased in patients with diabetic peripheral neuropathy, which can result in inflammation, exacerbated oxidative stress, nerve injury, and an insufficient blood supply [8]. As with retinopathy and neuropathy, the infiltration of inflammatory cells and expression of adhesion molecules and cytokines are detected in renal tissue obtained from subjects with DKD.

### 2.1. Macrophages

Blood monocytes and tissue macrophages are key members of the mononuclear phagocyte system, a component of innate immunity. Recently, intensive research has shown that the influx of macrophages is a prominent feature during the progression of chronic kidney disease [9] and is tightly correlated with the decline in the glomerular filtration rate (GFR), histological changes, and a poor outcome in this condition [10]. Macrophage-derived products, including reactive oxygen species (ROS), pro-inflammatory factors, metalloproteinases, and growth factors, can induce further renal damage in the setting of diabetes. Macrophage-depletion studies in rodent models have shown a causal role for macrophages in the progression of DKD. For instance, the deletion of a macrophage scavenger receptor protected diabetic mice from albuminuria, mesangial matrix expansion, and the overproduction of transforming growth factor β (TGF-β) [11]. The gene expression of pro-inflammatory factors was strongly suppressed in this diabetic mouse model.

Macrophages are classified into classically-activated M1 and alternatively-activated M2 states [12]. M1 macrophages promote the inflammatory process by elaborating pro-inflammatory factors and ROS, whereas M2 macrophages resolve inflammation and induce tissue remodeling with the release of growth factors. In simple inflammatory reactions, such as acute arthritis, macrophages progress linearly through the M1 and M2 phases. However, under chronic inflammatory conditions, such as atherosclerosis and DKD, both M1 and M2 macrophages coexist, and this imbalance in the M1/M2 macrophage phenotype may be a key point of DKD.

Notably, an experimental study showed that macrophages in streptozotocin-induced DKD are predominantly of the M1 phenotype [9,13]. Furthermore, RAW264.7 macrophages switch to the M1 phenotype when cultured in a high-glucose milieu. Consistent with these results, the glomerular expression of inducible nitric oxide and TNF-α is increased in insulin-deficient diabetic mouse models, suggesting the presence of M1 macrophages. The deletion of the Toll-like receptor-2 gene significantly induces a macrophage M1 to M2 polarization shift in the diabetic kidney, attenuates urinary albumin excretion, and protects podocytes from apoptosis and morphological changes [13]. The mechanisms by which M2 macrophages promote kidney repair and attenuate DKD progression are still under debate and merit further investigation.

### 2.2. Adhesion Molecules and Chemokines

While the precise molecular mechanisms that direct monocyte homing into the sites of renal inflammation are not fully understood, endothelial-leukocyte adhesion molecules, including vascular cell adhesion molecule 1 (VCAM1) and intercellular adhesion molecule 1 (ICAM1), have been considered to play important roles in the initiation of renal inflammation. Several studies provide compelling evidence supporting the importance of these molecules in monocyte recruitment. For example, these adhesion molecules are abundantly expressed in renal biopsy specimens obtained from patients with DKD. Furthermore, circulating levels of VCAM1 and ICAM1 are independently associated with DKD progression [14,15].

VCAM1, also known as CD106, is a transmembrane glycoprotein expressed in activated endothelium under a variety of pathologic conditions, including atherosclerosis and DKD. VCAM1 binds to α4β1 integrin, which is constitutively expressed on lymphocytes, monocytes, and eosinophils. The renal filtration function declines and urinary albumin excretion levels increase progressively with the elevation in VCAM1 levels in serum [16]. ICAM1, also known as CD54, is expressed structurally on endothelial cells and works as a ligand for lymphocyte function-associated antigen 1 (LFA-1) on monocytes. Its expression is also upregulated in endothelial cells in a diabetic milieu, and genetic polymorphisms in its gene are associated with DKD progression [17]. The deletion of the ICAM1 gene ameliorates renal inflammation in mice [18], indicating that ICAM1 contributes to the pathogenesis of DKD.

After adhering to the endothelium, monocytes migrate through endothelial cells via chemokines. Studies of DKD rodents and in human diabetic kidneys have shown that macrophages infiltrate the renal tissue in response to an upregulation of chemokines, such as monocyte chemoattractant protein 1 (MCP1), also known as CCL2. Nadkarni et al. investigated urinary MCP1 for its associations with the deterioration of the renal function in order to illuminate its potential as a urinary marker of DKD progression [19]. They reported that an increased level of urinary MCP1 was strongly associated with a decline in the renal function in patients with diabetes, suggesting that excreted MCP1 levels may be a useful biomarker for predicting progressive decline in DKD [20].

The induction of MCP1 by inflammatory cytokines in renal cells elicits the initial step of glomerular and tubular inflammation. Although a number of cytokines have been shown to be involved in the production of MCP1, there is compelling evidence that TNF-α functions as a potent inducer of MCP1 expression in the kidney. TNF-α is expressed in adipose tissue and has been implicated in the pathogenesis of insulin resistance and type 2 diabetes. In addition, it has been shown that the serum concentrations and urinary excretion of TNF-α are increased in patients with various kidney diseases, suggesting that excessive TNF-α in the systemic circulation may result in the upregulation of MCP1 in the kidney. Furthermore, elevated serum levels of TNF receptors (TNF-R) 1 or 2 are strongly associated with a risk of renal functional decline or ESRD [7,21]. Niewczas et al. investigated 194 circulating inflammatory factors in patients from three independent cohorts with type 1 and type 2 diabetes and demonstrated an extremely robust kidney risk inflammatory signature, including 17 proteins enriched in TNF-R superfamily members [22], which were associated with a 10-year risk of ESRD.

TNF-α secreted by infiltrated macrophages itself may also stimulate the local production of MCP1 in renal cells. Consequently, manipulating the MCP1 signaling axis offers an opportunity for therapeutic gain. A clinical trial is underway investigating the effectiveness of MCP1 inhibitors in patients with progressive DKD [23]. Treatment with emapticap pegol, a Spiegelmer that specifically binds and inhibits MCP1, was found to be generally safe and well tolerated. In that study, albuminuria was attenuated by treatment with emapticap pegol, and the beneficial effects were maintained even after cessation of the intervention.

Intrarenal amplification of macrophages also plays an important role in the progression of renal disease, including DKD. Colony-stimulating factor 1 (CSF1), also known as macrophage colony-stimulating factor, is a homodimer glycoprotein that governs the survival, proliferation, and differentiation of macrophages. In particular, CSF1 is required for macrophage proliferation throughout the G1 phase of the cell cycle, and this chemokine is constitutively expressed in glomerular mesangial cells, tubular epithelial cells, and endothelial cells. Experimental studies have demonstrated that the deletion of CSF1 in mice leads to the attenuation of macrophage recruitment and proliferation during renal inflammation [24]. Of interest, the implantation of CSF1-generating cells into the kidneys of autoimmune lupus mice incites local macrophage-mediated inflammation [25]. CSF1 is also upregulated in type 2 diabetic db/db mice [26]. Furthermore, the administration of an antibody against the CSF1 receptor attenuates the accumulation of macrophages in rodent models of renal diseases, including unilateral ureteric obstruction, renal allograft rejection, and DKD [27]. Given these findings, it is reasonable to suggest that CSF1 is a critical determinant of the survival and proliferation of macrophages in several kidney diseases.

Dysregulation of the immune system is an important determinant in the initiation of DKD. Studies have provided convincing evidence that T cells, B cells, and NK cells are critical drivers of inflammation. Activated T cells cause renal damage directly via cytotoxic effects and indirectly by the recruitment and activation of macrophages. It is possible that pro-inflammatory cytokines secreted by T cells activate neighboring macrophages and stimulate the production of MCP1 and CSF-1 from glomerular cells. B cells could affect the proliferation of Th17 and the production of pro-inflammatory cytokines in patients with diabetes. Both T cells and B cells were shown to infiltrate glomeruli in a diabetic mouse model of diabetes, however, the evidence for the involvement of B cells and NK cells in DKD is more limited than that for T cells [28].

Overall, these findings provide evidence that immune system components are involved in the initiation of DKD and that adhesion molecules and chemokines play an essential role in the progression of inflammation in DKD.

## 3. Signaling Cascade Governing Inflammatory Reactions

**Figure 1:**
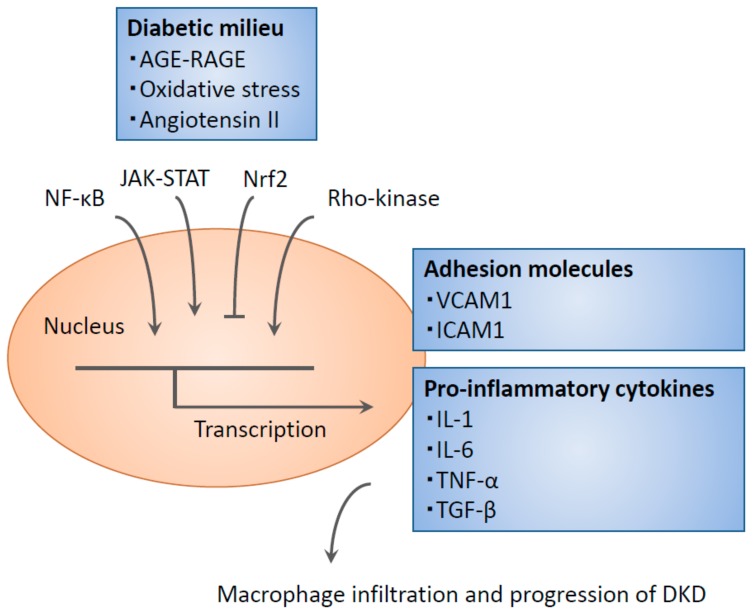
In the diabetic kidney, advanced glycation end products (AGEs) and oxidative stress activate a variety of signaling cascades to induce monocyte infiltration. In addition, chemokines drive inflammation, leading to macrophage-mediated tissue injury. DKD, Diabetic kidney disease; RAGE, receptor for AGE; VCAM1, vascular cell adhesion molecule 1; ICAM1, intercellular adhesion molecule 1; JAK-STAT, Janus kinase/signal transducer and activator of transcription; NF-κB, Nuclear factor Κb; Nrf2, nuclear factor-2 erythroid related factor 2; TNF-α, tumor necrosis factor α; TGF-β, transforming growth factor β.

A variety of upstream mediators (e.g., metabolic changes, altered redox balance, and intestinal dysbiosis) have been shown to be associated with inflammation in renal tissue. Cell death is induced under hyperglycemic conditions and activates the infiltration of macrophages and other immune cells into the kidney. Furthermore, the hyperglycemia-associated generation of advanced glycation end products (AGEs) and engagement of the receptor for AGE (RAGE) with its ligands induces oxidative stress and renal inflammation, resulting in a decline in renal function. Thus, the inhibition of AGE–RAGE signaling seems to be an attractive therapeutic strategy against renal inflammation. Interestingly, recent studies have reported the beneficial effects of RAGE blockade with FPS-ZM1 and RAGE-aptamers in DKD [29]. In addition to hyperglycemia-associated factors, systemic inflammation leads to dysregulation of the microcirculation network in the kidney and contributes to the in-site production of pro-inflammatory factors and ROS. Signaling events during renal inflammation orchestrate widespread transcriptional programs that affect the functions of adhesion molecules and inflammatory immune cells. An accumulating body of evidence exists defining the important roles for several signaling cascades in the control of renal inflammation, which we review below (Figure 1).

### 3.1. Nuclear Factor κB (NF-κB) and Activating Protein 1 (AP1)

Nuclear factor κB (NF-κB) is a family of transcription factors central in regulating inflammatory signals. The complex of NF-κB is a dimer of two members of the Rel family of proteins: p65 and p50. In unstimulated cells, NF-κB is sequestered in the cytoplasm by IκB family proteins, the best characterized of which is IκBα. IκBα phosphorylation leads to its ubiquitination and subsequent proteasome-mediated protein degradation, which exposes its nuclear localization signal. The subsequent recognition of NF-κB by karyopherin β directs it to the nuclear pore complex, where nuclear translocation takes place. In addition, the coactivator p300 can complex with the p65/p50 heterodimer to stabilize the chromatin structure for efficient transcription.

Glucose can activate NF-κB, resulting in increased inflammatory gene expression, in part through oxidative stress, AGEs, protein kinase C, and Mitogen-activated protein kinases (MAPKs) [30]. Increased renal NF-κB levels are detected in the kidneys of diabetic experimental models, and this activates glomerular and tubular cells to induce renal injury [26,31]. Downstream targets of NF-κB include adhesion molecules and pro-inflammatory cytokines (e.g., IL-6, TNF-α, MCP1, RANTES (Regulated on Activation, Normal T Cell Expressed and Secreted)), which all drive the development of DKD.

Similar to NF-κB, activating protein 1 (AP1) is activated by glucose and oxidative and inflammatory stimuli. AP1 regulates the TGF-β expression in mesangial cells, and the AP1 binding activity was found to be markedly enhanced by high glucose treatment [32]. PPARγ ligands are known to inhibit inflammatory gene expression via the attenuation of AP1, suggesting a potential anti-inflammatory therapeutic strategy by thiazolidine (TZD). Indeed, TZD decreases albuminuria, mesangial expansion, and the expression of TGF-β and osteopontin, a phosphor–glycoprotein adhesion molecule, in mouse models of diabetes [33]. Further clinical and mechanistic studies are required in order to validate the role of AP1 in the pathogenesis of DKD.

### 3.2. Janus Kinase/Signal Transducer and Activator of Transcription (JAK-STAT)

Experimental work over the past several years has shown the key roles of the Janus kinase/signal transducer and activator of transcription (JAK-STAT) pathway. The JAK-STAT pathway transduces signals from extracellular ligands (e.g., cytokines, chemokines, growth factors, and hormones) directly to the nucleus to activate a variety of cellular responses. Most of these reactions have been extensively studied in lymphoid cells, however, JAK-STAT signals also play critical roles in renal cells, including mesangial cells, podocytes, and tubular epithelial cells [34]. Studies have shown that JAK-STAT signaling is activated in renal tissue in patients with DKD. This pathway is activated by ROS induced by hyperglycemic states and is associated with glomerular hypertrophy [31]. A transcriptomic examination was performed in renal tissues obtained from subjects with early and progressive DKD [35]. In this analysis, all JAK-STAT genes were found to be highly expressed in the glomeruli from patients with early DKD compared with healthy controls. In contrast, tubular JAK-STAT genes were not increased in early DKD but were high in progressive DKD. Notably, the degree of induction of JAK-STAT gene expression was tightly and inversely correlated with the decline in the GFR.

A series of studies indicated that JAK family members JAK1, 2, and 3, as well as STAT1 and STAT3, are induced in DKD. In addition, Zhang et al. showed that the overexpression of JAK2 in podocytes can lead to worse renal damage in mouse models of diabetes [36]. Baricitinib, an oral inhibitor of the JAK family of protein tyrosine kinases that selectively inhibits JAK1 and JAK2, attenuates urinary albumin excretion in diabetic patients. Therefore, inhibiting JAK-STAT signaling may have potential utility for treating DKD and improving health outcomes in patients with diabetes [34].

### 3.3. Nuclear Factor-2 Erythroid Related Factor (Nrf2)-Keep1

The upregulation of Nrf2-dependent antioxidants attenuates systemic oxidative overload and renal inflammation. Beyond the resolution of oxidative stress, Nrf2 inhibits inflammation by directly regulating the transcription of pro-inflammatory cytokines (e.g., IL-1, IL-6). Kobayashi et al. reported that Nrf2 binds to the regulatory regions of inflammatory genes and thereby induces their transcription in macrophages and alleviates RNA polymerase II recruitment [37]. The pharmacological activation of Nrf2 decreases cytokine production, M1 macrophage accumulation, and the formation of an atherosclerotic plaque lipid core in the experimental model of streptozotocin-induced diabetic mice on an apolipoprotein E-deficient background [38]. Importantly, Nrf2 activation improves the pathological changes in the glomerulus of streptozotocin-injected diabetic mice through a reduction in oxidative stress, TGF-β expression, and extracellular matrix proteins [39]. Furthermore, Nrf2 attenuates mesangial hypertrophy induced by high glucose. Thus, Nrf2 activators have been suggested to prevent DKD. Bardoxolone methyl, one such Nrf2 activator, has been demonstrated in clinical trials to have reno-protective effects in patients with type 2 diabetes [40].

### 3.4. Rho-Kinase Signaling

A Rho-associated coiled-coil containing protein kinase (Rho-kinase) was initially identified as a regulator of Rho-induced stress fiber formation. Rho-kinase activation results in the phosphorylation of downstream targets, including myosin phosphatase target subunit, which has been demonstrated to mediate a wide array of cellular functions (e.g., cell proliferation, contraction, migration, and transcriptional regulation). Recent experimental studies have implicated Rho-kinase signaling in cardiovascular and kidney disease. Initial insights linking Rho-kinase to diabetes were gleaned from our studies defining Rho-kinase as a regulator of diabetic complications both in the microvasculature and large blood vessels. For example, Rho-kinase inhibition attenuates albuminuria, glomerular matrix expansion [41,42], and infiltration of macrophages [26] in mouse models of DKD. Subsequent analyses in endothelial cells identified Rho-kinase as a key molecule of vascular inflammation [43].

Of note, Rho-kinase mediates the production of MCP1 and monocyte chemotaxis toward glomerular cells. Furthermore, Rho-kinase functions as an important regulator of CSF1 production both in vitro and in vivo. From a transcriptional standpoint, there is evidence for an interaction between Rho-kinase and NF-κB activation. We recently showed that thrombin induces endothelial NF-κB activation through a molecular mechanism involving Rho-kinase [44]. Furthermore, Rho-kinase also plays an important role in the regulation of lysophosphatidic acid-induced endothelial NF-κB activation [45] and has been shown to be associated with neuropeptide-induced NF-κB activation and subsequent IL-8 induction in colonic epithelial cells [46]. Moreover, Rho exchange factor regulates NF-κB activation in monocytes, and the significance of the Rho-kinase/NF-κB axis in the kidney has been shown in mouse models of lipopolysaccharide injection [47]. These data suggest the existence of a mechanistic linkage between Rho-kinase and NF-κB signaling. CSF1 production in the glomerular mesangium is dependent on the NF-κB function, and the inhibition of Rho-kinase significantly inhibits NF-κB-mediated reporter activity via the p38 MAPK pathway. While some studies have demonstrated the direct regulation of Rho-kinase on either IκBα degradation or p65 phosphorylation, an interaction between Rho-kinase and the nuclear translocation of p65 without affecting either IκBα degradation or phosphorylation of p65 has also been reported [26].

Rho-kinase (ROCK) has two isoforms, ROCK1 and ROCK2, that share 65% sequence similarity but have different activation mechanisms. It has become increasingly clear that ROCK1 and ROCK2 play distinctive roles in regulating the cellular function. For instance, Takeda et al. showed the strong contribution of ROCK2 to the induction of E-selectin and MCP1 via NF-κB activation [48]. In vitro, ROCK2 is known to be localized in the nucleus and interacts with p300 acetyltransferase to activate p300-modulated transcription [49]. Taken together, these findings raise the possibility that targeting Rho-kinase may be a new therapeutic target against renal inflammation through a reduction in NF-κB-mediated macrophage accumulation.

## 4. Targeting Renal Inflammation for Prophylaxis and Therapy

With the discovery that inflammatory mediators are increased in DKD, researchers have begun to focus on therapeutic strategies targeting these inflammatory mediators. Several approaches have been proposed to treat inflammation in DKD, including lifestyle modifications, drugs, and dialysis optimization. As discussed above, the suppression of cytokine signaling with specific inhibitors, antibodies, or aptamers has the potential to reduce the risk of albuminuria and decline in the filtration function, highlighting the important role of these inflammatory mediators in DKD. In addition, novel pharmacological approaches to the management of diabetes have direct or indirect anti-inflammatory actions, the latter potentially attributable to an improvement in the metabolic status.

### 4.1. Sodium-Glucose Co-Transporter-2 (SGLT2) Inhibitors

SGLT2 inhibitors are now established therapies for treating hyperglycemia in patients with diabetes. By blocking SGLT2 at the proximal tubule, these agents limit the reabsorption of glucose, which in turn induces glycosuria and blood glucose reduction, independent of the insulin action [50]. Treatment with SGLT2 inhibitors leads to sustained systolic and diastolic blood pressure reductions, partially via natriuresis, which also contributes to the improvement of glomerular hyperfiltration and albuminuria through the activation of tubuloglomerular feedback [51].

Secondary outcome analyses in cardiovascular safety trials have shown the potential of SGLT2 inhibition to attenuate the risks of DKD progression and ESRD. For example, the renal composite outcomes (doubling of creatinine, renal replacement therapy, or renal death) decreased by 46% in patients with type 2 diabetes treated with empagliflozin [52]. A similar trend was reported with canagliflozin. The Canagliflozin and Renal Endpoints in Diabetes with Established Nephropathy Clinical Evaluation (CREDENCE) trial was designed to assess the efficacy and safety of canagliflozin versus placebo for reducing clinically important renal and cardiovascular outcomes in patients with diabetes and established kidney disease. This trial was restricted to participants who were taking the maximum tolerated dose of an angiotensin-converting enzyme inhibitor or angiotensin receptor blocker. Compared to a placebo, the reduction in the relative risk of renal-specific composites of ESRD, doubling of the creatinine level, or death from renal causes was greater in the canagliflozin group. An important observation from the CREDENCE trial is that the canagliflozin group also had a lower risk of cardiovascular death, myocardial infarction, or stroke and hospitalization for heart failure than the placebo group. Importantly, this trial showed no imbalance in rates of amputation or bone fracture, and no new safety concerns were identified [53]. Because few drugs are capable of preventing the progressive loss of the kidney function for this population, the current data will provide important evidence supporting the development of diabetes and nephrology guidelines.

Notably, canagliflozin reduces the plasma levels of TNF-R1 (9.2%; *p* < 0.001) and IL-6 (26.6%; *p* = 0.010) compared to glimepiride in patients with diabetes [54]. Similar effects were reported by Garvey et al. [55]. This evidence indicates that canagliflozin reverses molecular processes induced by inflammation. Experimental studies have also suggested the anti-inflammatory effects of SGLT2 inhibitors [56,57]. In diabetic Akita mice, empagliflozin inhibited albuminuria via a reduction in the levels of inflammatory cytokines, including MCP1 and IL-6 [57]. However, whether or not SGLT2 inhibitors can reduce the renal risk in patients with type 1 diabetes is unclear. This point will be investigated in a multicentre international randomized parallel group double-blind placebo-controlled clinical trial of EMPAgliflozin once daily to assess cardio-renal outcomes in patients with chronic KIDNEY disease (EMPA-KIDNEY), which will include patients with type 1 diabetes.

Basic mechanisms to explain anti-inflammatory effects may involve body weight loss and the improvement of obesity-induced insulin resistance. For instance, empagliflozin was shown to modulate energy metabolism by promoting fat utilization, browning of white adipose tissue, and blocking the infiltration of macrophages in high-fat-diet-fed obese mice [58]. Since there is a direct association between fat mass and the amount of pro-inflammatory mediators, a lower fat mass reflects less storage space for pro-inflammatory mediators and thus results in the attenuation of kidney inflammation. In addition, SGLT2 inhibitors may suppress inflammatory responses in the kidney by modulating RAS activity, hemodynamic changes, and changes in the immune system function. The increase in ketone bodies and inhibition of oxidative stress might also have contributed to the beneficial effects on renal inflammation.

Further studies are required in order to assess the inflammatory markers and elucidate the specific contribution of SGLT2 inhibition to the reduction in the DKD risk and associated mortality. When considered alongside very recent observations concerning the SGLT2-mediated oxidative pathway in mesangial cells [59], it will also be interesting to explore whether or not SGLT2 inhibitors affect glomerular inflammation directly.

### 4.2. Glucagon-Like Peptide 1 (GLP-1) Receptor Agonists and Dipeptidyl Peptidase 4 (DPP-4) Inhibitors

Glucagon-like peptide 1 (GLP-1) is a gut-derived peptide secreted from intestinal L-cells upon meal ingestion, that regulates glucose homeostasis by modulating the pancreatic islet cell function, food intake, and gastrointestinal motility. In addition to pancreatic β cells, GLP-1 receptor is expressed in proximal tubular cells and the kidney vasculature. GLP-1 receptor-mediated natriuresis is induced by the inhibition of sodium-hydrogen exchanger 3 (NHE3), which is located at the brush border of the renal proximal tubule. Mechanistically, GLP-1 receptor agonists (GLP-1RA) promote the phosphorylation of NHE3 at Ser552 and Ser605, which results in a decreased activity of NHE3 and natriuresis. Experimental studies in rodents and humans have shown that the activation of GLP-1 receptor leads to an increase in lithium clearance and pH, indicating the existence of NHE3-mediated regulation. Such proximal natriuresis would be expected to activate tubuloglomerular feedback and afferent vasoconstriction, leading to the downregulation of the renal blood flow and GFR. However, there were no changes in the renal hemodynamics or GFR in subjects with type 2 diabetes treated with GLP-1RA. These data are attributed to the direct nitric oxide-dependent reduction of afferent vascular resistance by GLP-1RA, which might override vasoconstriction induced by tubuloglomerular feedback [60].

GLP-1 receptors are expressed in renal tissue as well as the pancreas, heart, and intestine. Although the exact distribution of GLP-1 receptors in the kidney is not well characterized, clinical studies have shown that treatment with GLP-1RA reduces new-onset macroalbuminuria. These renal benefits may be explained by the known actions of GLP-1RA on metabolic risk factors for DKD, including reductions in blood glucose, blood pressure, and weight. However, several studies in rodents have demonstrated renoprotective effects of GLP-1RA beyond metabolic improvements in models of DKD. Aside from GLP-1RA, DPP-4 inhibitors also exert various extrapancreatic actions. DPP-4 inhibitors can attenuate inflammatory signaling pathways. For example, linagliptin exerts antioxidant and anti-inflammatory actions in endothelium, independent of its glucose-lowering effects [61,62]. In addition, circulating levels of inflammatory cytokines and markers of inflammation were reduced in diabetic patients treated with sitagliptin or vildagliptin [63]. Experimental studies have demonstrated the renoprotective effects of DPP-4 inhibitors in the models of DKD and nondiabetic glomerular injury [64]. Some of these beneficial actions may be due to elevated GLP-1 levels. However, evidence suggests that the albuminuria-lowering effect of the DPP-4 inhibitor is weak compared to that of GLP-1RA [65,66].

The protective effect of GLP-1RA is mediated at least in part via inhibitory effects on renal inflammation, as well as NHE3-dependent sodium reabsorption [67]. For instance, exenatide was shown to inhibit albuminuria and the influx of glomerular macrophages in type 2 diabetic db/db mice [68]. Albuminuria and mesangial expansion were attenuated by treatment with exenatide in streptozotocin-injected type 1 diabetic rats through a mechanism involving inflammatory cytokines and adhesion molecules [69]. Furthermore, exenatide and dulaglutide reduced the circulating levels of C-reactive protein (CRP) in patients with type 2 diabetes [70]. Studies have shown that GLP-1RA’s anti-inflammatory effects are modulated through the prevention of oxidative stress. Subsequent detailed investigations have revealed that liraglutide treatment inhibited albuminuria and nicotinamide adenine dinucleotide phosphate oxidase activity in a KK/Ta-Akita mouse model of type 1 diabetes without altering the blood glucose levels [71]. A more detailed understanding will be required to fully characterize the exact renal tissue distribution of GLP-1 receptors, as well as the associated anti-inflammatory effects and antioxidant mechanisms, in order to explain the renoprotection mediated by GLP-1RA.

### 4.3. Metformin

Metformin is now widely used for the treatment of type 2 diabetes as a first-line drug. Clinical studies have demonstrated that metformin not only attenuates chronic inflammation through the improvement of metabolic parameters (e.g., hyperglycemia, dyslipidemia), but also has direct anti-inflammatory properties. Metformin regulates miR-34a, a tumor-suppressor microRNA, to inhibit mesangial inflammation under high glucose conditions [72]. By activating AMPK, metformin disrupts the phosphorylation of STAT signaling and attenuates the differentiation of monocytes into macrophages [73].

### 4.4. Immunosuppressive Agents

Pharmacological modulation of the immune system may have beneficial effects on DKD development. For example, glucocorticoids are known to exert anti-inflammatory and immunosuppressive actions by genomic and nongenomic effects. Glucocorticoids have been extensively used in nephrotic syndrome. Cell-based studies suggest that glucocorticoids may have protective effects on podocyte injury [74]. Mechanistically, glucocorticoids reduce podocyte apoptosis and increase the number of podocyte progenitors via key components of the glucocorticoid receptor complex (e.g., heat shock protein 90, immunophilins FK506 binding protein (FKBP) 51, and FKBP52). However, the risk and potential clinical benefits of glucocorticoids are different for each patient. It is important to consider whether the benefits outweigh the side effects.

Mycophenolate mofetil is an anti-lymphocyte agent with immunosuppressive properties. This drug is clinically used to prevent allograft rejection. Mycophenolic acid, the active metabolite of mycophenolate mofetil, attenuates lymphocyte proliferation by blocking the early phases of the cell cycle. Experimental studies have demonstrated that mycophenolate mofetil decreases proteinuria and glomerulosclerosis through the reduction of macrophage infiltration in rodent models of type 1 and type 2 diabetes [75,76]. Furthermore, podocyte apoptosis and ROS production were attenuated in mycophenolate mofetil-treated diabetic mice [77]. Clinical evidence is required before recommendations can be made for treatment against DKD.

Regulatory T-cells (Tregs) control self-tolerance and allogeneic tolerance, and the dysfunction of Tregs is supposed to mediate the progression of DKD. Recent evidence from the Treg inhibition approach with anti-CD25 antibody emphasizes the importance of Treg in DKD. Treatment with anti-CD25 accelerated kidney damage in db/db mice and the adoptive transfer of Tregs inhibited proteinuria and glomerular hypertrophy [78]. Therefore, Tregs may be an attractive target for the treatment of DKD. Since the transfer of Treg has technical limitations, approaches that enhance endogenous Tregs are likely to be beneficial for DKD patients.

## 5. Conclusions and Future Perspectives

It is becoming clear that DKD is associated with the increased expression of inflammation-associated mediators. Since the diagnosis of DKD is still difficult due to a lack of reliable biomarkers capable of predicting patient outcomes, especially in the early stages (albuminuria is not specific for diabetic renal damage), the levels of pro-inflammatory cytokines may be a useful marker for making a diagnosis of DKD. From this standpoint, urinary proteomics and peptidomics have gained attention as a study tool for the detection of diagnostic markers of DKD. The urinary omics studies in DKD have revealed several markers, including inflammatory mediators such as MCP1 and TGF-β [79,80]. Whether inflammation is a trigger or a result of a chronic underlying condition is an important topic. Gene manipulation studies on the impact of renal inflammation on DKD development have greatly expanded our knowledge in this field. Furthermore, studies exploring the cause of this pro-inflammatory milieu and the contribution of these pathways to DKD development will strengthen the significance of inflammatory mediators as biomarker of DKD.

Recent systems biological approaches have revealed molecular abnormalities in DKD and have directly led to the identification of potential targets for DKD. A comprehensive transcriptome analysis demonstrated that the top differentially regulated genes in DKD tissue are associated with inflammation and fibrosis [81]. In this assessment, the complement signal was the highest degree of statistical significance in both glomeruli and tubulointerstitium. Studies will be required to determine the pathological significance of the complement system in human DKD.

The cumulative data on the role of inflammation in the pathogenesis of DKD are sufficient to warrant their consideration as therapeutic targets. Targeting inflammatory cytokines or adhesion molecules will offer novel avenues for therapeutic intervention. However, a central challenge remains: how to leverage our comprehension of the inflammatory mechanism in order to establish an intervention that can prevent DKD progression and prolong the lifespan of diabetic patients. Over the past decade, new classes of anti-hyperglycemic drugs with renal benefits have been introduced for the management of diabetes, namely SGLT2 inhibitors and GLP-1RA. These agents seem to have additional pleiotropic anti-inflammatory properties mediated through distinct molecular mechanisms by both direct and indirect actions. A greater understanding of the mechanisms underlying these agents’ renal benefits would facilitate the development of novel therapies for DKD and its associated sequelae.

## References

[1] Adler A.I., Stevens R.J., Manley S.E., Bilous R.W., Cull C.A., Holman R.R., UKPDS GROUP (2003). Development and progression of nephropathy in type 2 diabetes: The United Kingdom Prospective Diabetes Study (UKPDS 64). Kidney Int..

[2] Goldfine A.B., Shoelson S.E. (2017). Therapeutic approaches targeting inflammation for diabetes and associated cardiovascular risk. J. Clin. Invest..

[3] Simo-Servat O., Simo R., Hernandez C. (2016). Circulating Biomarkers of Diabetic Retinopathy: An Overview Based on Physiopathology. J. Diabetes Res..

[4] Paeschke S., Paeschke S., Baum P., Toyka K.V., Blüher M., Koj S., Klöting N., Bechmann I., Thiery J., Kosacka J. (2019). The Role of Iron and Nerve Inflammation in Diabetes Mellitus Type 2-Induced Peripheral Neuropathy. Neuroscience.

[5] Xu L., Lin X., Guan M., Zeng Y., Liu Y. (2019). Verapamil Attenuated Prediabetic Neuropathy in High-Fat Diet-Fed Mice through Inhibiting TXNIP-Mediated Apoptosis and Inflammation. Oxid Med. Cell Longev..

[6] Doupis J., Lyons T.E., Wu S., Gnardellis C., Dinh T., Veves A. (2009). Microvascular reactivity and inflammatory cytokines in painful and painless peripheral diabetic neuropathy. J. Clin. Endocrinol Metab..

[7] Pop-Busui R., Ang L., Holmes C., Gallagher K., Feldman E.L. (2016). Inflammation as a Therapeutic Target for Diabetic Neuropathies. Curr. Diab. Rep..

[8] Ganesh Yerra V., Negi G., Sharma S.S., Kumar A. (2013). Potential therapeutic effects of the simultaneous targeting of the Nrf2 and NF-kappaB pathways in diabetic neuropathy. Redox Biol..

[9] Tian S., Chen S.Y. (2015). Macrophage polarization in kidney diseases. Macrophage (Houst).

[10] Klessens C.Q.F., Zandbergen M., Wolterbeek R., Bruijn J.A., Rabelink T.J., Bajema I.M., IJpelaar D.H.T. (2017). Macrophages in diabetic nephropathy in patients with type 2 diabetes. Nephrol. Dial. Transpl..

[11] Usui H.K., Shikata K., Sasaki M., Okada S., Matsuda M., Shikata Y., Ogawa D., Kido Y., Nagase R., Yozai K. (2007). Macrophage scavenger receptor-a-deficient mice are resistant against diabetic nephropathy through amelioration of microinflammation. Diabetes.

[12] Landis R.C., Quimby K.R., Greenidge A.R. (2018). M1/M2 Macrophages in Diabetic Nephropathy: Nrf2/HO-1 as Therapeutic Targets. Curr. Pharm. Des..

[13] Devaraj S., Tobias P., Kasinath B.S., Ramsamooj R., Afify A., Jialal I. (2011). Knockout of toll-like receptor-2 attenuates both the proinflammatory state of diabetes and incipient diabetic nephropathy. Arterioscler Thromb. Vasc. Biol..

[14] Clausen P., Jacobsen P., Rossing K., Jensen J.S., Parving H.H., Feldt-Rasmussen B. (2000). Plasma concentrations of VCAM-1 and ICAM-1 are elevated in patients with Type 1 diabetes mellitus with microalbuminuria and overt nephropathy. Diabet Med..

[15] Hojs R., Ekart R., Bevc S., Hojs N. (2016). Markers of Inflammation and Oxidative Stress in the Development and Progression of Renal Disease in Diabetic Patients. Nephron.

[16] Liu J.J., Yeoh L.Y., Sum C.F., Tavintharan S., Ng X.W., Liu S., Lee S.B., Tang W.E., Lim S.C., SMART2D study (2015). Vascular cell adhesion molecule-1, but not intercellular adhesion molecule-1, is associated with diabetic kidney disease in Asians with type 2 diabetes. J. Diabetes Complicat..

[17] Lim A.K., Tesch G.H. (2012). Inflammation in diabetic nephropathy. Mediators Inflamm..

[18] Okada S., Shikata K., Matsuda M., Ogawa D., Usui H., Kido Y., Nagase R., Wada J., Shikata Y., Makino H. (2003). Intercellular adhesion molecule-1-deficient mice are resistant against renal injury after induction of diabetes. Diabetes.

[19] Nadkarni G.N., Rao V., Ismail-Beigi F., Fonseca V.A., Shah S.V., Simonson M.S., Cantley L., Devarajan P., Parikh C.R., Coca S.G. (2016). Association of Urinary Biomarkers of Inflammation, Injury, and Fibrosis with Renal Function Decline: The ACCORD Trial. Clin. J. Am. Soc. Nephrol..

[20] Satirapoj B., Dispan R., Radinahamed P., Kitiyakara C. (2018). Urinary epidermal growth factor, monocyte chemoattractant protein-1 or their ratio as predictors for rapid loss of renal function in type 2 diabetic patients with diabetic kidney disease. BMC Nephrol..

[21] Niewczas M.A., Gohda T., Skupien J., Smiles A.M., Walker W.H., Rosetti F., Cullere X., Eckfeldt J.H., Doria A., Mayadas T.N. (2012). Circulating TNF receptors 1 and 2 predict ESRD in type 2 diabetes. J. Am. Soc. Nephrol..

[22] Niewczas M.A., Pavkov M.E., Skupien J., Smiles A., Md Dom Z.I., Wilson J.M., Park J., Nair V., Schlafly A., Saulnier P.J. (2019). A signature of circulating inflammatory proteins and development of end-stage renal disease in diabetes. Nat. Med..

[23] Menne J., Eulberg D., Beyer D., Baumann M., Saudek F., Valkusz Z., Więcek A., Haller H., Emapticap Study Group (2017). C-C motif-ligand 2 inhibition with emapticap pegol (NOX-E36) in type 2 diabetic patients with albuminuria. Nephrol. Dial. Transplant..

[24] Lenda D.M., Kikawada E., Stanley E.R., Kelley V.R. (2003). Reduced macrophage recruitment, proliferation, and activation in colony-stimulating factor-1-deficient mice results in decreased tubular apoptosis during renal inflammation. J. Immunol..

[25] Naito T., Yokoyama H., Moore K.J., Dranoff G., Mulligan R.C., Kelley V.R. (1996). Macrophage growth factors introduced into the kidney initiate renal injury. Mol. Med..

[26] Matoba K., Kawanami D., Tsukamoto M., Kinoshita J., Ito T., Ishizawa S., Kanazawa Y., Yokota T., Murai N., Matsufuji S. (2014). Rho-kinase regulation of TNF-alpha-induced nuclear translocation of NF-kappaB RelA/p65 and M-CSF expression via p38 MAPK in mesangial cells. Am. J. Physiol. Renal Physiol..

[27] Lim A.K., Ma F.Y., Nikolic-Paterson D.J., Thomas M.C., Hurst L.A., Tesch G.H. (2009). Antibody blockade of c-fms suppresses the progression of inflammation and injury in early diabetic nephropathy in obese db/db mice. Diabetologia.

[28] Pichler R., Afkarian M., Dieter B.P., Tuttle K.R. (2017). Immunity and inflammation in diabetic kidney disease: Translating mechanisms to biomarkers and treatment targets. Am. J. Physiol. Renal Physiol..

[29] Sanajou D., Ghorbani Haghjo A., Argani H., Aslani S. (2018). AGE-RAGE axis blockade in diabetic nephropathy: Current status and future directions. Eur. J. Pharmacol..

[30] Pérez-Morales R.E., Del Pino M.D., Valdivielso J.M., Ortiz A., Mora-Fernández C., Navarro-González J.F. (2018). Inflammation in Diabetic Kidney Disease. Nephron.

[31] Toth-Manikowski S., Atta M.G. (2015). Diabetic Kidney Disease: Pathophysiology and Therapeutic Targets. J. Diabetes Res..

[32] Weigert C., Sauer U., Brodbeck K., Pfeiffer A., Häring H.U., Schleicher E.D. (2000). AP-1 proteins mediate hyperglycemia-induced activation of the human TGF-beta1 promoter in mesangial cells. J. Am. Soc. Nephrol..

[33] Nicholas S.B., Liu J., Kim J., Ren Y., Collins A.R., Nguyen L., Hsueh W.A. (2010). Critical role for osteopontin in diabetic nephropathy. Kidney Int..

[34] Tuttle K.R., Brosius III F.C., Adler S.G., Kretzler M., Mehta R.L., Tumlin J.A., Tanaka Y., Haneda M., Liu J., Silk M.E. (2018). JAK1/JAK2 inhibition by baricitinib in diabetic kidney disease: Results from a Phase 2 randomized controlled clinical trial. Nephrol. Dial. Transplant..

[35] Brosius F.C., Ju W. (2018). The Promise of Systems Biology for Diabetic Kidney Disease. Adv. Chronic Kidney Dis..

[36] Zhang H., Nair V., Saha J., Atkins K.B., Hodgin J.B., Saunders T.L., Myers M.G.Jr., Werner T., Kretzler M., Brosius F.C. (2017). Podocyte-specific JAK2 overexpression worsens diabetic kidney disease in mice. Kidney Int..

[37] Kobayashi E.H., Suzuki T., Funayama R., Nagashima T., Hayashi M., Sekine H., Tanaka N., Moriguchi T., Motohashi H., Nakayama K. (2016). Nrf2 suppresses macrophage inflammatory response by blocking proinflammatory cytokine transcription. Nat. Commun..

[38] Lazaro I., Lopez-Sanz L., Bernal S., Oguiza A., Recio C., Melgar A., Jimenez-Castilla L., Egido J., Madrigal-Matute J., Gomez-Guerrero C. (2018). Nrf2 Activation Provides Atheroprotection in Diabetic Mice Through Concerted Upregulation of Antioxidant, Anti-inflammatory, and Autophagy Mechanisms. Front. Pharmacol..

[39] Zheng H., Whitman S.A., Wu W., Wondrak G.T., Wong P.K., Fang D., Zhang D.D. (2011). Therapeutic potential of Nrf2 activators in streptozotocin-induced diabetic nephropathy. Diabetes.

[40] Pergola P.E., Raskin P., Toto R.D., Meyer C.J., Huff J.W., Grossman E.B., Krauth M., Ruiz S., Audhya P., Christ-Schmidt H. (2011). Bardoxolone methyl and kidney function in CKD with type 2 diabetes. N. Engl. J. Med..

[41] Matoba K., Kawanami D., Okada R., Tsukamoto M., Kinoshita J., Ito T., Ishizawa S., Kanazawa Y., Yokota T., Murai N. (2013). Rho-kinase inhibition prevents the progression of diabetic nephropathy by downregulating hypoxia-inducible factor 1alpha. Kidney Int..

[42] Kawanami D., Matoba K., Utsunomiya K. (2016). Signaling pathways in diabetic nephropathy. Histol. Histopathol..

[43] Kawanami D., Matoba K., Okada R., Tsukamoto M., Kinoshita J., Ishizawa S., Kanazawa Y., Yokota T., Utsunomiya K. (2013). Fasudil inhibits ER stress-induced VCAM-1 expression by modulating unfolded protein response in endothelial cells. Biochem. Biophys. Res. Commun..

[44] Kawanami D., Matoba K., Kanazawa Y., Ishizawa S., Yokota T., Utsunomiya K. (2011). Thrombin induces MCP-1 expression through Rho-kinase and subsequent p38MAPK/NF-kappaB signaling pathway activation in vascular endothelial cells. Biochem. Biophys. Res. Commun..

[45] Shimada H., Rajagopalan L.E. (2010). Rho kinase-2 activation in human endothelial cells drives lysophosphatidic acid-mediated expression of cell adhesion molecules via NF-kappaB p65. J. Biol. Chem..

[46] Zhao D., Kuhnt-Moore S., Zeng H., Wu J.S., Moyer M.P., Pothoulakis C. (2003). Neurotensin stimulates IL-8 expression in human colonic epithelial cells through Rho GTPase-mediated NF-kappa B pathways. Am. J. Physiol. Cell Physiol..

[47] Meyer-Schwesinger C., Dehde S., von Ruffer C., Gatzemeier S., Klug P., Wenzel U.O., Stahl R.A., Thaiss F., Meyer T.N. (2009). Rho kinase inhibition attenuates LPS-induced renal failure in mice in part by attenuation of NF-kappaB p65 signaling. Am. J. Physiol. Renal Physiol..

[48] Takeda Y., Matoba K., Kawanami D., Nagai Y., Akamine T., Ishizawa S., Kanazawa Y., Yokota T., Utsunomiya K. (2019). ROCK2 Regulates Monocyte Migration and Cell to Cell Adhesion in Vascular Endothelial Cells. Int. J. Mol. Sci..

[49] Tanaka T., Nishimura D., Wu R.C., Amano M., Iso T., Kedes L., Nishida H., Kaibuchi K., Hamamori Y. (2006). Nuclear Rho kinase, ROCK2, targets p300 acetyltransferase. J. Biol. Chem..

[50] Thomas M.C., Cherney D.Z.I. (2018). The actions of SGLT2 inhibitors on metabolism, renal function and blood pressure. Diabetologia.

[51] Kawanami D., Matoba K., Takeda Y., Nagai Y., Akamine T., Yokota T., Sango K., Utsunomiya K. (2017). SGLT2 Inhibitors as a Therapeutic Option for Diabetic Nephropathy. Int. J. Mol. Sci..

[52] Wanner C., Inzucchi S.E., Lachin J.M., Fitchett D., von Eynatten M., Mattheus M., Johansen O.E., Woerle H.J., Broedl U.C., Zinman B. (2016). Empagliflozin and Progression of Kidney Disease in Type 2 Diabetes. N. Engl. J. Med..

[53] Perkovic V., Jardine M.J., Neal B., Bompoint S., Heerspink H.J.L., Charytan D.M., Edwards R., Agarwal R., Bakris G., Bull S. (2019). Canagliflozin and Renal Outcomes in Type 2 Diabetes and Nephropathy. N. Engl. J. Med..

[54] Heerspink H.J.L., Perco P., Mulder S., Leierer J., Hansen M.K., Heinzel A., Mayer G. (2019). Canagliflozin reduces inflammation and fibrosis biomarkers: A potential mechanism of action for beneficial effects of SGLT2 inhibitors in diabetic kidney disease. Diabetologia.

[55] Garvey W.T., Van Gaal L., Leiter L.A., Vijapurkar U., List J., Cuddihy R., Ren J., Davies M.J. (2018). Effects of canagliflozin versus glimepiride on adipokines and inflammatory biomarkers in type 2 diabetes. Metabolism.

[56] Tahara A., Takasu T., Yokono M., Imamura M., Kurosaki E. (2017). Characterization and comparison of SGLT2 inhibitors: Part 3. Effects on diabetic complications in type 2 diabetic mice. Eur. J. Pharmacol..

[57] Vallon V., Gerasimova M., Rose M.A., Masuda T., Satriano J., Mayoux E., Koepsell H., Thomson SC., Rieg T. (2014). SGLT2 inhibitor empagliflozin reduces renal growth and albuminuria in proportion to hyperglycemia and prevents glomerular hyperfiltration in diabetic Akita mice. Am. J. Physiol. Renal Physiol..

[58] Xu L., Nagata N., Nagashimada M., Zhuge F., Ni Y., Chen G., Mayoux E., Kaneko S., Ota T. (2017). SGLT2 Inhibition by Empagliflozin Promotes Fat Utilization and Browning and Attenuates Inflammation and Insulin Resistance by Polarizing M2 Macrophages in Diet-induced Obese Mice. EBioMedicine.

[59] Maki T., Maeno S., Maeda Y., Yamato M., Sonoda N., Ogawa Y., Wakisaka M., Inoguchi T. (2019). Amelioration of diabetic nephropathy by SGLT2 inhibitors independent of its glucose-lowering effect: A possible role of SGLT2 in mesangial cells. Sci. Rep..

[60] Muskiet M.H., Tonneijck L., Smits M.M., Kramer M.H., Diamant M., Joles J.A., van Raalte D.H. (2016). Acute renal haemodynamic effects of glucagon-like peptide-1 receptor agonist exenatide in healthy overweight men. Diabetes Obes. Metab..

[61] Kröller-Schön S., Knorr M., Hausding M., Oelze M., Schuff A., Schell R., Sudowe S., Scholz A., Daub S., Karbach S. (2012). Glucose-independent improvement of vascular dysfunction in experimental sepsis by dipeptidyl-peptidase 4 inhibition. Cardiovasc Res..

[62] Kanasaki K. (2018). The role of renal dipeptidyl peptidase-4 in kidney disease: Renal effects of dipeptidyl peptidase-4 inhibitors with a focus on linagliptin. Clin. Sci..

[63] Barbieri M., Rizzo M.R., Marfella R., Boccardi V., Esposito A., Pansini A., Paolisso G. (2013). Decreased carotid atherosclerotic process by control of daily acute glucose fluctuations in diabetic patients treated by DPP-IV inhibitors. Atherosclerosis.

[64] Higashijima Y., Tanaka T., Yamaguchi J., Tanaka S., Nangaku M. (2015). Anti-inflammatory role of DPP-4 inhibitors in a nondiabetic model of glomerular injury. Am. J. Physiol. Renal Physiol..

[65] Pollock C., Stefánsson B., Reyner D., Rossing P., Sjöström C.D., Wheeler D.C., Langkilde A.M., Heerspink H.J.L. (2019). Albuminuria-lowering effect of dapagliflozin alone and in combination with saxagliptin and effect of dapagliflozin and saxagliptin on glycaemic control in patients with type 2 diabetes and chronic kidney disease (DELIGHT): A randomised, double-blind, placebo-controlled trial. Lancet Diabetes Endocrinol..

[66] Rosenstock J., Perkovic V., Johansen O.E., Cooper M.E., Kahn S.E., Marx N., Alexander J.H., Pencina M., Toto R.D., Wanner C. (2019). Effect of Linagliptin vs Placebo on Major Cardiovascular Events in Adults With Type 2 Diabetes and High Cardiovascular and Renal Risk: The CARMELINA Randomized Clinical Trial. JAMA..

[67] Kawanami D., Matoba K., Sango K., Utsunomiya K. (2016). Incretin-Based Therapies for Diabetic Complications: Basic Mechanisms and Clinical Evidence. Int. J. Mol. Sci..

[68] Park C.W., Kim H.W., Ko S.H., Lim J.H., Ryu G.R., Chung H.W., Han S.W., Shin S.J., Bang B.K., Breyer M.D. (2017). Long-term treatment of glucagon-like peptide-1 analog exendin-4 ameliorates diabetic nephropathy through improving metabolic anomalies in db/db mice. J. Am. Soc. Nephrol..

[69] Kodera R., Shikata K., Kataoka H.U., Takatsuka T., Miyamoto S., Sasaki M., Kajitani N., Nishishita S., Sarai K., Hirota D. (2011). Glucagon-like peptide-1 receptor agonist ameliorates renal injury through its anti-inflammatory action without lowering blood glucose level in a rat model of type 1 diabetes. Diabetologia.

[70] Ferdinand K.C., White W.B., Calhoun D.A., Lonn E.M., Sager P.T., Brunelle R., Jiang H.H., Threlkeld R.J., Robertson K.E., Geiger M.J. (2014). Effects of the once-weekly glucagon-like peptide-1 receptor agonist dulaglutide on ambulatory blood pressure and heart rate in patients with type 2 diabetes mellitus. Hypertension.

[71] Fujita H., Morii T., Fujishima H., Sato T., Shimizu T., Hosoba M., Tsukiyama K., Narita T., Takahashi T., Drucker D.J. (2014). The protective roles of GLP-1R signaling in diabetic nephropathy: Possible mechanism and therapeutic potential. Kidney Int..

[72] Wu C., Qin N., Ren H., Yang M., Liu S., Wang Q. (2018). Metformin Regulating miR-34a Pathway to Inhibit Egr1 in Rat Mesangial Cells Cultured with High Glucose. Int. J. Endocrinol..

[73] Vasamsetti S.B., Karnewar S., Kanugula A.K., Thatipalli A.R., Kumar J.M., Kotamraju S. (2015). Metformin inhibits monocyte-to-macrophage differentiation via AMPK-mediated inhibition of STAT3 activation: Potential role in atherosclerosis. Diabetes.

[74] Guess A., Agrawal S., Wei C.C., Ransom R.F., Benndorf R., Smoyer W.E. (2010). Dose- and time-dependent glucocorticoid receptor signaling in podocytes. Am. J. Physiol. Renal Physiol..

[75] Utimura R., Fujihara C.K., Mattar A.L., Malheiros D.M., Noronha I.L., Zatz R. (2003). Mycophenolate mofetil prevents the development of glomerular injury in experimental diabetes. Kidney Int..

[76] Rodríguez-Iturbe B., Quiroz Y., Shahkarami A., Li Z., Vaziri N.D. (2005). Mycophenolate mofetil ameliorates nephropathy in the obese Zucker rat. Kidney Int..

[77] Seo J.W., Kim Y.G., Lee S.H., Lee A., Kim D.J., Jeong K.H., Lee K.H., Hwang S.J., Woo J.S., Lim S.J. (2015). Mycophenolate Mofetil Ameliorates Diabetic Nephropathy in db/db Mice. Biomed. Res. Int..

[78] Eller K., Kirsch A., Wolf A.M., Sopper S., Tagwerker A., Stanzl U., Wolf D., Patsch W., Rosenkranz A.R., Eller P. (2011). Potential role of regulatory T cells in reversing obesity-linked insulin resistance and diabetic nephropathy. Diabetes.

[79] Pena M.J., Mischak H., Heerspink H.J. (2016). Proteomics for prediction of disease progression and response to therapy in diabetic kidney disease. Diabetologia.

[80] Verhave J.C., Bouchard J., Goupil R., Pichette V., Brachemi S., Madore F., Troyanov S. (2013). Clinical value of inflammatory urinary biomarkers in overt diabetic nephropathy: A prospective study. Diabetes Res. Clin. Pract..

[81] Woroniecka K.I., Park A.S., Mohtat D., Thomas D.B., Pullman J.M., Susztak K. (2011). Transcriptome analysis of human diabetic kidney disease. Diabetes.

